# Expression levels of the JAK/STAT pathway in the transition from hormone-sensitive to hormone-refractory prostate cancer

**DOI:** 10.1038/sj.bjc.6603871

**Published:** 2007-06-26

**Authors:** L Tam, L M McGlynn, P Traynor, R Mukherjee, J M S Bartlett, J Edwards

**Affiliations:** 1 Section of Surgical and Translational Sciences, Division of Cancer Sciences and Molecular Pathology, Glasgow Royal Infirmary, Glasgow G31 2ER, UK

**Keywords:** prostate, IL-6, IL-6R, JAK, STAT, hormone refractory

## Abstract

The main cause of prostate cancer-related mortality is the development of hormone-refractory disease. Circulating serum levels of IL-6 are raised in hormone-refractory prostate cancer patients and evidence from cell line studies suggests that the IL-6R/JAK/STAT3 pathway may be involved in development of this disease. In the current study we investigate if expression levels of these family members are implicated in the development of hormone-refractory prostate cancer. Immunohistochemistry using IL-6R, JAK1, STAT3, pSTAT3^Tyr705^ and pSTAT3^Ser727^ antibodies was performed on 50 matched hormone-sensitive and hormone-refractory tumours pairs. An increase in expression of cytoplasmic IL-6 receptor, with the development of hormone-refractory prostate cancer was associated with reduced time to relapse (*P*=0.0074) while an increase in expression of cytoplasmic pSTAT3^Tyr705^ was associated with reduced patient survival (*P*=0.0003). In addition, those patients with high expression of cytoplasmic pSTAT3^Tyr705^ in their hormone-refractory tumours had significantly shorter time to death from biochemical relapse and overall survival in comparison to those patients with low expression of cytoplasmic pSTAT3^Tyr705^ (*P*=0.002 and *P*=0.0027, respectively). Activation of STAT3, via phosphorylation is associated with reduced patient survival, suggesting that activation of the IL-6R/JAK/STAT3 pathway is involved with development of hormone-refractory prostate cancer.

Carcinoma of the prostate (CaP) is an increasing healthcare problem. In the UK prostate cancer is the most common male malignancy and is the second main cause of cancer-related deaths among men. The majority of prostate cancer patients present with locally advanced or metastatic disease, which may be treated using androgen ablation therapy. Response rates to androgen ablation therapy are initially high (70–80%), however most patients relapse with resistance to androgen ablation within 18–24 months, termed as developing hormone-refractory prostate cancer (Beemsterboer *et al*, 1999). The lack of effective therapies directed against hormone-refractory prostate cancer is related to the poor understanding of the molecular mechanisms that drive progression to this refractory state (McEwan, 2004).

One possible mechanism underlying the development of hormone-refractory prostate cancer is upregulation of the IL-6 receptor/JAK/STAT3 cascade. As prostate cancer progresses from hormone sensitive to hormonerefractory, the circulating concentrations of IL-6 in the serum of patients increase (Fearon *et al*, 1991; Drachenberg *et al*, 1999; Heinrich *et al*, 2003). It is postulated that this results in activation of the IL-6 receptor/JAK/STAT3 cascade (Hobisch *et al*, 1998), which has previously been reported to increase androgen receptor (AR) activity (Culig, 2003). Indeed, the increase in proliferation rate observed in prostate cancer cell line models in response to IL-6 has been demonstrate to be via activation of STAT3 (Dhir *et al*, 2002; Godoy-Tundidor *et al*, 2005; Sanford and Dewille, 2005). In addition, *in vitro* studies have demonstrated that IL-6-dependent activation of the JAK/STAT3 pathway is accompanied by transition from hormone-sensitive to hormone-insensitive prostate cancer cell growth (Lee *et al*, 2003). LNCaP cells, normally undergo apoptosis when androgens are withdrawn, however treatment with IL-6 or transfection with constitutively active STAT3 results in protection of the cells from apoptosis and therefore resistance to androgen deprivation (Lee *et al*, 2004). Inhibition of STAT3 activation results in the induction of apoptosis in cells even in the presence of IL-6 (Barton *et al*, 2001, 2004). The hypothesis that STAT3 is involved in the development of hormone-refractory prostate cancer is further supported by the observation that levels of activated STAT3 are significantly higher in AR-negative cells (DU145 and PC3) than in AR-positive (LNCaP cells) (Mora *et al*, 2002). STAT3 activation may therefore act to promote cell growth and survival in hormone-refractory prostate cancer independent of the AR.

Conversely, STAT3 has been implicated in the development of hormone-refractory disease via interaction with the AR (Culig, 2003, 2004). In LNCaP cells the activated dimer of STAT3 binds ligand-free AR in the cytoplasm before entering the nucleus, facilitating the translocation of the AR in to the nucleus in the absence of androgens (Matsuda *et al*, 2001; Chen *et al*, 2002). Functional cell line studies demonstrate that the AR/STAT-3 complex can promote androgen-regulated gene transcription even in the absence of androgens (Chen *et al*, 2002; Trachtenberg and Blackledge, 2002; Yamamoto *et al*, 2003). This mechanism is supported by data that demonstrate IL-6 can activate the AR in a ligand-independent manner (Ueda *et al*, 2002; Corcoran and Costello, 2003). Evidence in clinical tissue to support these *in vitro* observations are sparse, although it is reported that IL-6 receptor expression is eightfold higher in prostate cancer tissue compared to normal tissue (Giri *et al*, 2001) and that phosphorylated STAT3 is observed in 82% of human prostate tumours and expression levels correlate with Gleason score (Barton *et al*, 2004).

In summary, despite the large number of *in vitro* functional reports implying a role of IL-6R/JAK/STAT3 pathway in prostate cancer progression there appears to be little data confirming the role of this pathway in the development of clinical hormone-refractory prostate cancer. This study investigates both the expression levels and activation of the IL-6R/JAK/STAT3 pathway in matched hormone-sensitive and hormone-refractory tumours from the same patient. This will enable us to assess if changes in expression and activation of pathway members are associated with development of hormone-refractory prostate cancer. Therefore, we aim to identify whether inhibition of this pathway would lead to improved patient outcome after progression to hormone-refractory prostate cancer.

## MATERIALS AND METHODS

### Patients

Fifty patients were retrospectively selected for this study. Ethical approval was obtained from the Multi-centre Research Ethics Committee (MREC Scotland) and local research ethics committees. Inclusion criteria for this study were that each patient was required to have both hormone-sensitive and hormone-refractory tumours available for analysis. Tumours were defined as hormone sensitive if PSA fell by at least 50% during hormone treatment and subsequently hormone refractory if two consecutive rises in serum PSA of >10% was observed during hormone therapy.

### Immunohistochemistry

All antibodies used in this study had specificity confirmed by western blotting and on paraffin-embedded cell pellets known to express the proteins of interest (LNCaP and MCF-7 cells). IHC was performed on 5 *μ*m, archival formalin-fixed paraffin-embedded prostate tumour sections. Two methods of antigen retrieval were used, sections were microwaved under pressure (15 psi) in TE solution (5 mM Tris base, pH 8.0 and 1 mM sodium EDTA (JAK1, 3332; pSTAT3^Tyr705^, 9131; and pSTAT3^Ser727^, 9134, Cell Signaling Technology) or incubated in 10 mM citrate buffer (epitope retrieval solution × 10, DakoCytomation, Glostrup, Denmark) in a calibrated water bath at 96°C for 20 min (IL-6R, C20, SC-661, Santa Cruz and STAT3, 9132, Cell Signaling Technology). Non-specific background staining was blocked using 1.5% (v v^−1^*)* normal horse serum in tri-phosphate buffered saline and incubated for 20 min at room temperature. All antibodies were incubated overnight at 4°C. The concentrations used for each antibody were as follows: IL-6R 1 : 500, JAK1 1 : 100, STAT3 1 : 100, pSTAT3^Tyr705^ 1 : 50 and pSTAT3^Ser727^ 1 : 50. Staining was developed using the LSAB plus kit (DakoCytomation) and chromagen was detected using 3,3'diaminobenzidine (Vector Labs, UK). A positive and negative control slide was included in each IHC run, negative controls were incubated in an isotype-matched control antibody at a concentration of 1 mg ml^−1^.

### Scoring criteria

Tissue staining was scored blind by two independent observers using a weighted histoscore method (Kirkegaard *et al*, 2006), also known as the H score system (McCarty *et al*, 1986). The full tissue section was examined and expression score calculated as follows (1 × % cells staining weakly positive)+(2 × % cells staining moderately positive)+(3 × % cells staining strongly positive). Maximum score was 300. An interclass correlation coefficient (ICCC) for each protein was calculated by SPSS for Windows to confirm consistency between observers and the mean of the two observers' scores were used for analysis. ICCC of greater than 0.7 is considered as excellent (Kirkegaard *et al*, 2006).

### Statistical analysis

Statistical analysis was performed using SPSS for Windows. Descriptive analysis was used on variables such as age at diagnosis, serum PSA (pre- and post-relapse), Gleason sum, time to biochemical relapse, time to death from biochemical relapse and overall survival. Median and inter-quartile (IQR) ranges were calculated from these analyses. To determine if there was a change in expression in progression from hormone-sensitive to hormone-refractory disease a Wilcoxon signed-rank test was used to compare the hormone-sensitive expression score to the hormone-refractory expression score for each protein and each location. Survival analysis was performed using Kaplan–Meier curves and the log-rank test, low expression was defined as an expression score less than or equal to the median and high expression as expression score greater than the median. A change in expression level in hormone-sensitive and hormone refractory-matched tumours was defined as the mean difference between the expression scores that each observer assigns for protein expression plus two standard deviations. The number of histoscore units defined as a change in expression for each individual protein is shown in Table 1.

## RESULTS

### Patient characteristics

Fifty pairs of hormone-sensitive and hormone-refractory prostate cancer tumours were analysed. The median age at diagnosis was 70 (IQR 64–73) years and the median PSA at diagnosis was 24.6 (IQR 6.4–79.8)ng  ml^−1^. The median time to biochemical relapse was 2.55 (IQR 1.55–5.24) years, the median time to death from hormone relapse was 1.49 (IQR 0.98–2.14) years, and the median overall survival was 5.82 (IQR 3.03–6.78) years. The median Gleason sum was 8 (IQR 6–9) for hormone-sensitive tumours and 9 (IQR 8–9) for hormone-refractory tumours. The range of Gleason sum for both tumour types was 2–10. All patients received chemical or surgical castration and 39 also received anti-androgens.

### Protein expression

The variation in observer scoring was calculated by ICCC. An ICCC of 0.7 is classed as excellent and an ICCC of 1 indicates identical scores. All scorer variations assessed in this study by ICCC consistently achieved an ICCC of 0.7 or above, ICCC values for each protein at each cellular location is given in Table 1.

**Figure 1:**
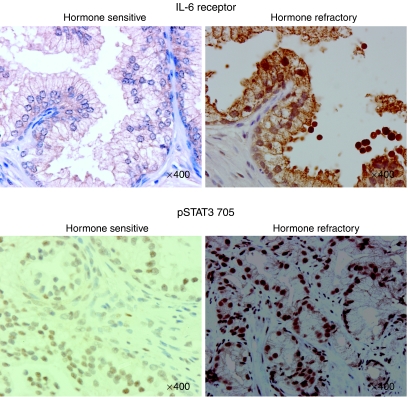
Images of matched hormone-sensitive and hormone-refractory prostate tumours whose expression increased in the transition from hormone-sensitive to hormone-refractory disease (upper panel: IL-6 receptor and lower panel: pSTAT3^Tyr705^). Positive staining is brown in colour and is indicated by arrows according to their location, M, membrane; C, cytoplasm; and N, nucleus. Counterstaining is blue and is represented in the stroma (S).

Membrane and cytoplasmic expression was observed for IL-6 receptor, while only cytoplasmic expression was observed for JAK1, and STAT3. Cytoplasmic and nuclear expression was seen for both phosphorylated STAT3 proteins. An example of an increase in expression for IL-6 receptor and pSTAT3^Tyr705^ in the transition from hormone-sensitive to hormone-refractory disease is shown in Figure 1.

### Protein expression levels in hormone-sensitive and hormone-refractory tumours

**Figure 2:**
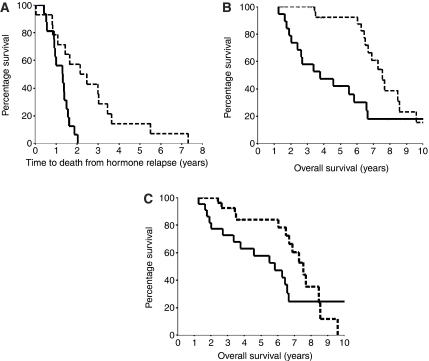
(**A**) Kaplan–Meier plot comparing time to death from biochemical relapse for those patients with hormone-refractory tumours with high cytoplasmic pSTAT3^Tyr705^ expression (solid line) (27 patients) compared to those patients with hormone-refractory tumours with low cytoplasmic pSTAT3^Tyr705^ expression (broken line) (23 patients) (*P*=0.002, hazard ratio 4.2 (95% CI 1.59–11.34)). (**B**) Kaplan–Meier plot comparing overall survival for those patients with hormone-refractory tumours with high-cytoplasmic pSTAT3^Tyr705^ expression (solid line) (19 patients) compared to those patients with hormone-refractory tumours with low cytoplasmic pSTAT3^Tyr705^ expression (broken line) (31 patients) (*P*=0.0027, hazard ratio 2.87 (95% CI 1.39–5.92)). (**C**) Kaplan–Meier plot for high-pSTAT3^Tyr705^ protein expression in the nucleus *vs* low nuclear pSTAT3^Tyr705^ protein expression in hormone-refractory prostate tumours. Those patients with high pSTAT3^Tyr705^ (solid line) (22 patients) expression had shorter overall survival compared to those with low pSTAT3^Tyr705^ (broken line) (28 patients); however this was not a significant change (*P*=0.25).

To assess if protein expression levels were associated with clinical endpoints (relapse and survival), Kaplan–Meier graphs were plotted for tumours expressing low levels of specific proteins compared to high levels. Those patients that expressed high levels of cytoplasmic pSTAT3^Tyr705^ in their hormone-refractory tumour had significantly shorter time to death from biochemical relapse and than those patients with low cytoplasmic pSTAT3^Tyr705^ expression (*P*=0.002, hazard ratio 4.25 (95% CI 1.59–11.34)) (Figure 2A). This also translated into significantly shorter overall survival (*P*=0.0027, hazard ratio 2.87 (95% CI 1.39–5.92)) (Figure 2B). The median overall survival in those patients whose tumours expressed high levels was 3.77 (IQR 1.11–6.43) years compared to 7.55 (IQR 6.69–8.41) years for those whose tumours expressed low levels. A trend with overall survival was also noted for nuclear pSTAT3^Tyr705^ in hormone-refractory prostate cancer; however due to the lines crossing after 8 years this was not significant (*P*=0.250) (Figure 2C). The median overall survival for those patients whose tumours had low expression was 7.49 (IQR 6.52–8.57) years compared to 5.82 (IQR 3.49–8.15) years for those patients whose tumours expressed high levels of nuclear pSTAT3^Tyr705^. Expression levels of all other proteins in hormone-sensitive or hormone-refractory tumours were not associated with time to relapse, time to death from relapse, or overall survival. When pSTAT3^Tyr705^ and pSTAT3^Ser727^ expression levels were divided by Gleason (<7, 7 or >7) no change in expression was observed in the cytoplasm or the nucleus. In addition the ratio of pSTAT3^Tyr705^ and pSTAT3^Ser727^ does not correlate with Gleason.

### Changes in protein expression with the development of hormone-refractory prostate cancer

**Figure 3:**
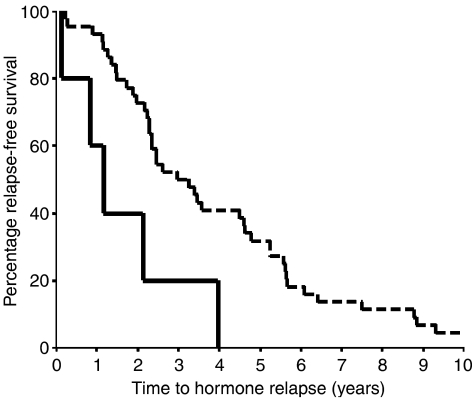
Kaplan–Meier plot for an increase in IL-6R protein expression *vs* no change or a decrease in IL-6R protein expression in the transition from hormone-sensitive to hormone-refractory disease. Those patients with an increase in IL-6R expression (solid line) (six patients) had significantly shorter time to biochemical relapse compared to those with no change or a decrease in IL-6R expression (broken line) (45 patients) (*P*=0.0076, hazard ratio 3.45 (95% CI 1.31–9.07)).

An increase in cytoplasmic IL-6 receptor expression from hormone sensitive to hormone refractory was associated with reduced time to biochemical relapse (*P*=0.0074) (Figure 3). The median time to relapse for patients whose tumours had a decrease or no change in expression with the development of hormone-refractory prostate cancer was 2.97 (IQR 1.89–4.07) year compared to 1.18 (IQR 0.45–1.92) years for those patients whose tumours exhibited a rise in expression.

**Figure 4:**
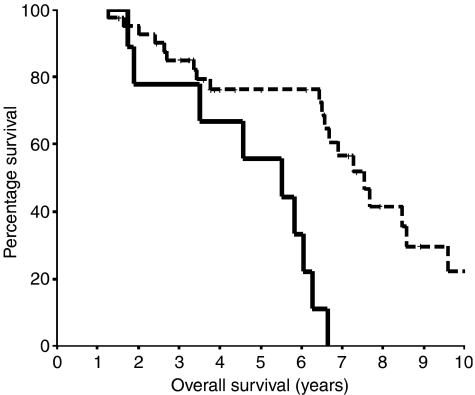
Kaplan–Meier plot for an increase in pSTAT3^Tyr705^ protein expression in the cytoplasm *vs* no change or a decrease in pSTAT3^Tyr705^ protein expression in the transition from hormone-sensitive to hormone-refractory disease. Those patients with an increase in pSTAT3^Tyr705^ (solid line) (nine patients) expression had significantly shorter overall survival compared to those with no change or a decrease in pSTAT3^Tyr705^ expression (broken line) (50 patients) (*P*=0.0003, hazard ratio 4.52 (95% CI 1.85–11.52)).

An increase in expression of cytoplasmic pSTAT3^Tyr705^ with progression to hormone-refractory prostate cancer was also associated with a reduction in overall survival (*P*=0.0003, hazard ratio 4.52 (95% CI 1.85–11.52)) (Figure 4A). The median overall survival for those patients whose tumours exhibited a decrease or no change in expression was 7.54 (IQR 6.52–8.57) years compared to 5.51 (IQR 2.77–8.26) years for those patients whose tumours exhibited a rise in expression. Changes in the expression levels of all the other proteins investigated in the transition from hormone-sensitive to hormone-refractory tumours were not significantly associated with time to relapse, time to death from relapse, or overall survival.

## DISCUSSION

Approximately 50% of patients with advanced prostate cancer have elevated levels of serum IL-6 in comparison with men with normal prostates, benign prostatic hyperplasia, prostatitis and localised disease (Twillie *et al*, 1995; Drachenberg *et al*, 1999). In addition IL-6 has been associated with progression from hormone-sensitive to hormone-insensitive disease in animal models via interaction with AR cofactors (Wallner *et al*, 2006). One possible mode by which IL-6 may influence progression of prostate cancer to the hormone-refractory state is by activating the IL-6 receptor/JAK1/STAT3 pathway, resulting in differentiation and inhibition of apoptosis (Spiotto and Chung, 2000a; Smith *et al*, 2001). Over-expression of IL-6 receptor in androgen-sensitive human LNCaP cells results in the conversion to androgen-independent growth both *in vitro* and *in vivo* (Lee *et al*, 2003) and depletion of IL-6 results in decreased proliferation of hormone-insensitive cells but not hormone-sensitive cells (Lou *et al*, 2000).

The IL-6 receptor is predominantly located in the cell membrane (Lou *et al*, 2000), however the IL-6/IL-6 receptor complex may be taken into the cell by endocytosis as part of the protein-recycling process (Nesbitt and Fuller, 1992). This is the cell's method of downregulating receptors once the ligand has produced the appropriate signal (Nesbitt and Fuller, 1992). Therefore as the IL-6 receptor, unlike the other signalling proteins in the pathway does not have a phosphorylated form, the localisation of the IL-6 receptor to the cytoplasm may be used as a surrogate marker for activation. This study demonstrates that there is an association with an increase in cytoplasmic IL-6 receptor expression with development of hormone-refractory prostate cancer and time to biochemical relapse. However these results should be treated with caution as only 12% (six patients) of the patients in our cohort exhibited this increase. We would therefore aim to increase our cohort size to confirm these results.

JAK1 and STAT3 are proteins that become activated in sequence, upon the binding of IL-6 to the IL-6 receptor (Schindler and Darnell, 1995). STAT3 is phosphorylated at two different sites, tyrosine 705 position and serine 727. The tyrosine kinase, JAK1 phosphorylates STAT3 at tyrosine 705, while the kinase(s) that mediate serine phosphorylation remain to be determined, evidence suggests that mitogen-activated protein kinase may be responsible (Briscoe *et al*, 1996a, 1996b). Immunohistochemical staining in our cohort of patients has demonstrated that both phosphorylated forms of STAT3, pSTAT3^Tyr705^ and pSTAT3^Ser727^ are found in the cytoplasmic and nuclear compartments of the cell. STAT3 dimerisation occurs in the cytoplasm before it enters the nucleus (Corcoran and Costello, 2003), the presence of activated STAT3 in the cytoplasm, therefore, provides a ‘snapshot’ of activated STAT3 before it enters the nucleus. Although it has been previously reported that it is serine phosphorylation at 727 that modulates the DNA binding and/or transcriptional activity of STAT3 dimers (Dhir *et al*, 2002) our data shows no evidence of pSTAT3^Ser727^ being associated with development of hormone-refractory prostate cancer. However, it has been reported that phosphorylation of STAT3 at serine 727 via ERK1 and/or 2 negatively regulates STAT3 activity (Kim *et al*, 2002; Tian *et al*, 2004), this supports our data that pSTAT3^Ser727^ is not associated with the development of hormone-refractory prostate cancer or prostate cancer patient survival.

There does however appear to be a significant role for pSTAT3^Tyr705^ in the progression to hormone-refractory prostate cancer. High expression of cytoplasmic pSTAT3^Tyr705^ in hormone-refractory prostate cancer tissue is associated with quicker time to death from hormone relapse and shorter overall survival. In addition, an increase in pSTAT3^Tyr705^ with the development of hormone-refractory prostate cancer is associated with shorter overall survival. These results fit with the hypothesis that activation of the IL-6 receptor/JAK1/STAT3 pathway is involved in the development of hormone-refractory prostate cancer. However, nuclear expression of pSTAT3^Tyr705^ expression was not associated with clinical parameters in this study. This may be due to the fact that although phosphorylation of tyrosine 705 increases STAT3 activity it is not sufficient to induce its relocation from the cytoplasm to the nucleus. Dimerisation of STAT3 is required for its translocation to the nucleus and this is regulated by reversible acetylation of a single lysine (Lys) residue (position 685) (Yuan *et al*, 2005). More research is therefore required to understand the exact mechanisms for activation and translocation of STAT3 to the nucleus and also the consequence of STAT3 phosphorylation in clinical prostate cancer tissue. Cell line studies demonstrate that STAT3 activation results in an increase in proliferation and induces neuroendocrine differentiation, although out with the scope of the current study these parameters warrant further investigation in clinical tissue to establish the route by which STAT3 influences prostate cancer patient survival (Spiotto and Chung, 2000b).

In summary, these data support the hypothesis that the IL-6 receptor/JAK1/STAT3 pathway is activated in the progression of hormone-refractory prostate cancer. Cytoplasmic expression of IL-6 receptor and pSTAT3^Tyr705^ are associated with reduced time to biochemical relapse and reduced time to death from hormone relapse respectively, therefore, supporting the strategy for targeting this pathway in hormone-refractory prostate cancer treatments. A recent report demonstrates that this pathway can be targeted and successfully inhibited in other disease using a humanised monoclonal antibody that targets the IL-6 receptor (in a similar to which herceptin targets HER2 (Jia *et al*, 2004; Nakahara and Nishimoto, 2006; Nishimoto and Kishimoto, 2006) and this approach has proved successful in prostate cancer animal models (Wallner *et al*, 2006). Use of this drug (tocilixumab) in phase II clinical trials for rheumatoid arthritis has proved the clinical benefit of IL-6 blockade and we suggest that such a strategy should be applied to hormone-refractory (Nishimoto and Kishimoto, 2006).

## Figures and Tables

**Table 1:** The expression scores that represent a change in expression levels in the transition from hormone-sensitive to hormone-refractory disease

	**Mean difference+2 s.d.**	**ICCC**
IL-6R (membrane)	67.4	0.85
IL-6R (cytoplasmic)	33.7	0.83
JAK1	25.3	0.81
STAT3	32.5	0.86
pSTAT3^Tyr 705^ (cytoplasmic)	31.5	0.70
pSTAT3^Tyr 705^ (nuclear)	64.4	0.87
pSTAT3^Ser727^ (cytoplasmic)	18.7	0.93
pSTAT3^Ser727^ (nuclear)	54.9	0.87

These were calculated from the mean observer difference plus 2 standard deviations. The inter- class correlation coefficients are also shown demonstrating consistent scoring.
